# Function of Selective Neuromodulatory Projections in the Mammalian Cerebral Cortex: Comparison Between Cholinergic and Noradrenergic Systems

**DOI:** 10.3389/fncir.2018.00047

**Published:** 2018-06-22

**Authors:** Hee-Jun Rho, Jae-Hyun Kim, Seung-Hee Lee

**Affiliations:** Sensory Processing Laboratory, Department of Biological Sciences, Korea Advanced Institute of Science & Technology (KAIST), Daejeon, South Korea

**Keywords:** neuromodulation, cerebral cortex, cholinergic projection, noradrenergic projection, basal forebrain, locus coeruleus

## Abstract

Cortical processing is dynamically modulated by different neuromodulators. Neuromodulation of the cerebral cortex is crucial for maintaining cognitive brain functions such as perception, attention and learning. However, we do not fully understand how neuromodulatory projections are organized in the cerebral cortex to exert various functions. The basal forebrain (BF) cholinergic projection and the locus coeruleus (LC) noradrenergic projection are well-known neuromodulatory projections to the cortex. Decades of studies have identified anatomical and physiological characteristics of these circuits. While both cholinergic and noradrenergic neurons widely project to the cortex, they exhibit different levels of selectivity. Here, we summarize their anatomical and physiological features, highlighting selectivity and specificity of these circuits to different cortical regions. We discuss the importance of selective modulation by comparing their functions in the cortex. We highlight key features in the input-output circuits and target selectivity of these neuromodulatory projections and their roles in controlling four major brain functions: attention, reinforcement, learning and memory, sleep and wakefulness.

## Introduction

The cerebral cortex is divided into distinct areas that compute specific sensory, motor, or other cognitive information. As the cortex develops into a wide and thick structure, each sub-region of the cortex can work as a module. Depending on the task demand, an animal needs to devote a particular cortical region to process specific information. Neuromodulatory inputs to the cortex are known to play important roles in guiding the transition of cortical processing (McCormick, 1992; Hasselmo, 1995; Gu, 2002; Lee and Dan, 2012). Neuromodulation of the proper cortical region is critical for an animal to perform optimal behaviors (Hasselmo, 1995; Harris and Thiele, 2011; Lee and Dan, 2012). For example, attention modulates a subset of cortical modules that receive and process the attended stimuli selectively. In contrast, global modulation of the cortex is more important for the transition from sleep to wakefulness. How does this cortical modulation occur in distinct patterns in different brain states? To answer this, we need to explore how neuromodulatory projections are organized in the cortex. The mammalian brain has an increased capacity and performs many cognitive functions. Accompanying the larger brain, is a larger cerebral cortex with thick cortical layers and complex circuits. Mediating transitions in cortical processing is a complicated multi-modal function, thus necessitating an intricate structure of neuromodulatory projections.

**Figure 1:**
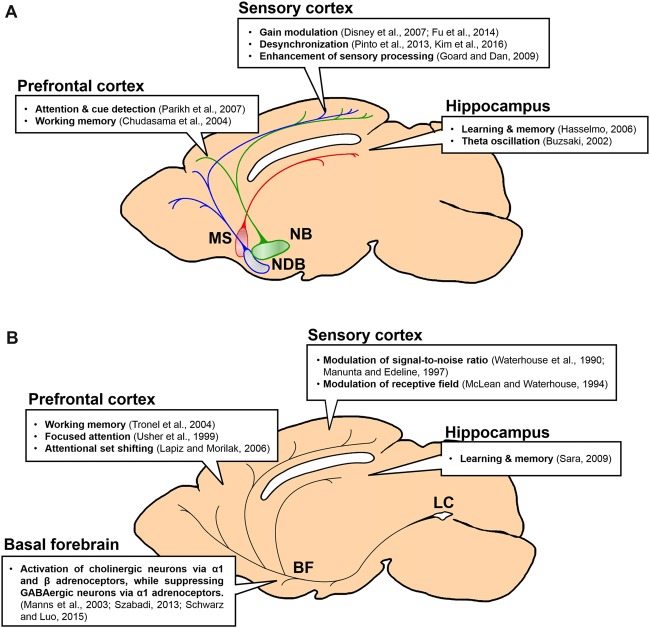
Projections and functions of basal forebrain (BF) cholinergic and locus coeruleus (LC) noradrenergic neurons to forebrain regions. Function of each projection is summarized in a box. **(A)** Projections of BF cholinergic neurons to the prefrontal cortex (PFC), the sensory cortex and the hippocampus. Green, the nucleus basalis (NB) and its projection; blue, the horizontal diagonal band nucleus (HDB) and its projection; red, the medial septal nucleus (MS) and its projection. **(B)** Projections of LC noradrenergic neurons to the BF, the PFC, the sensory cortex and the hippocampus.

Among the many neuromodulatory projections, cholinergic and noradrenergic inputs to the cerebral cortex have been studied extensively. Both neuromodulators are critical for cognitive behaviors in mammals, such as attention, arousal, learning and memory (Hasselmo, 1999, 2006; Sara, 2009; Sarter et al., 2009; Sara and Bouret, 2012; Schwarz and Luo, 2015; Ballinger et al., 2016). Interestingly, within the cortex, these two neuromodulatory systems show distinct characteristics in their anatomical and physiological features, even though they have common target regions from prefrontal to sensory cortices (Loughlin et al., 1986; Woolf, 1991). Here, we summarize and compare the anatomical and functional features of cholinergic and noradrenergic projections in the cortex (Figure 1). We first discuss how selective these projections are in terms of their axonal divergence in the cortex, target cell and receptor types. We further compare input convergence to the cholinergic and the noradrenergic systems and their mutual connectivity. At the end, we examine important functions of these two modulatory systems in relation to the selectivity of their projections to the cortex.

## Acetylcholine

### Anatomical Organization of the Cholinergic System

Central cholinergic systems in the mammalian brain are largely divided into the basal forebrain (BF) and the midbrain cholinergic nuclei. The BF cholinergic neurons send projections to the entire cerebral cortex in both primates and rodents (Mesulam et al., 1983; Rye et al., 1984; Woolf, 1991). The BF encompasses several nuclei of the medial septal nucleus (MS), the vertical diagonal band nucleus (VDB), the horizontal diagonal band nucleus (HDB), the nucleus basalis (NB) and the substantia innominata (SI). Different nuclei send cholinergic projections to distinct cortical areas. For example, VDB cholinergic neurons project to the medial part of the cortex including the cingulate and retrosplenial cortex (Rye et al., 1984; Woolf, 1991; Mechawar et al., 2000). The HDB sends cholinergic projections to the cingulate, the retrosplenial, the entorhinal, the perirhinal and the visual cortex (Rye et al., 1984; Woolf, 1991; Kim et al., 2016). The medial and rostral parts of the NB project to the cingulate and the somatosensory cortex, and the posterior part projects to the temporal area and auditory cortex (Woolf, 1991; Kim et al., 2016; Chavez and Zaborszky, 2017). The HDB cholinergic neurons mainly project to the visual cortex, while the anterior and posterior parts of the NB project to the somatosensory and the auditory cortex, respectively (Eggermann et al., 2014; Kim et al., 2016). Thus, BF cholinergic projections to the cortex seem to be topographically segregated within the selective projection to the discrete area in the cortex.

**Figure 2:**
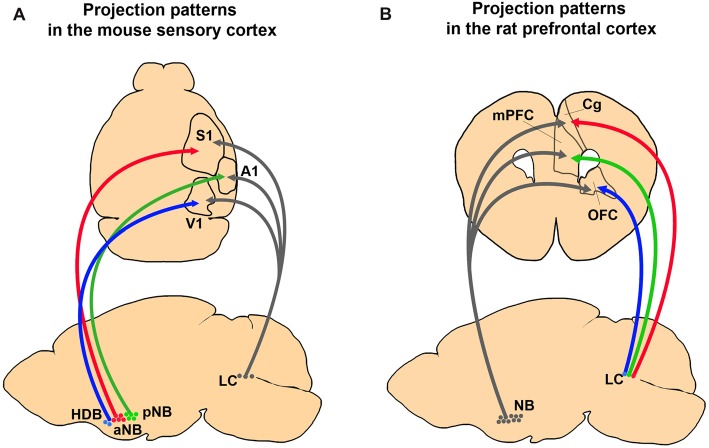
Comparing anatomical selectivity of BF cholinergic and LC noradrenergic projections to the cortex in rodents.** (A)** Projection patterns of BF cholinergic and LC noradrenergic projections to the primary sensory cortices in a mouse brain. The neurons in the NB and the HDB show selective innervation to the primary sensory cortices whereas LC neurons show diverging innervation (Kim et al., 2016). **(B)** Projection patterns of the BF and the LC neurons to the PFC in a rat brain. The NB neurons send more diverging projections than the LC neurons (Chandler et al., 2013).

The selective projection of BF cholinergic neurons has a high potential for selective modulation of the cortex. For example, it has been shown that acetylcholine (ACh) concentration in the sensory cortex of the anesthetized rat increases when the animal receives sensory stimuli whereas ACh concentration in the medial prefrontal cortex (mPFC) does not show any changes (Fournier et al., 2004). On the other hand, when the animal performs a detection task that requires high levels of attention, ACh concentration increases selectively in the mPFC but not in the motor cortex (Parikh et al., 2007). These results suggest the sensory stimuli can drive cholinergic neurons that innervate a particular sensory cortex, while the top-down attention to the potential stimuli can drive the cholinergic neurons that innervate the mPFC. Interestingly, the cholinergic projection to the PFC is less selective than the cholinergic projection to the sensory cortices (Figure 2). The sensory cortex receives modality-selective inputs from the BF cholinergic neurons (Kim et al., 2016). On the contrary, more than 80% of the NB cholinergic neurons project to multiple areas in the PFC including the anterior cingulate cortex (ACC), the mPFC, and the orbitofrontal cortex (OFC; Chandler et al., 2013). However, this study did not examine the projection of cholinergic neurons in other BF nuclei, and it is possible that the anterior BF nuclei such as the VDB or the HDB might show selective projection to the sub-regions in the PFC (Gaykema et al., 1990).

### Cholinergic Transmission in the Cerebral Cortex

There are two modes of cholinergic transmission in the cortex. One is the classical synaptic transmission, which mediates specific and tight modulation of the postsynaptic neurons. The other is the volume transmission, which can occur more globally and slowly in the cortex. It is still controversial which type of transmission is predominant in the cortex (Sarter et al., 2009; Ballinger et al., 2016). Although the *en passant* axonal boutons of cholinergic neurons can mediate volume transmission broadly in the cortex, acetylcholinesterase (AChE) restricts the diffusion of ACh by enzymatic hydrolysis after the release (Sarter et al., 2009). Indeed, the ACh concentration is elevated by 60 times in AChE knock-out mice compared to the wild-type mice (Hartmann et al., 2007). Thus, the cholinergic transmission in the cortex can be highly selective within the local cortical region.

**Figure 3:**
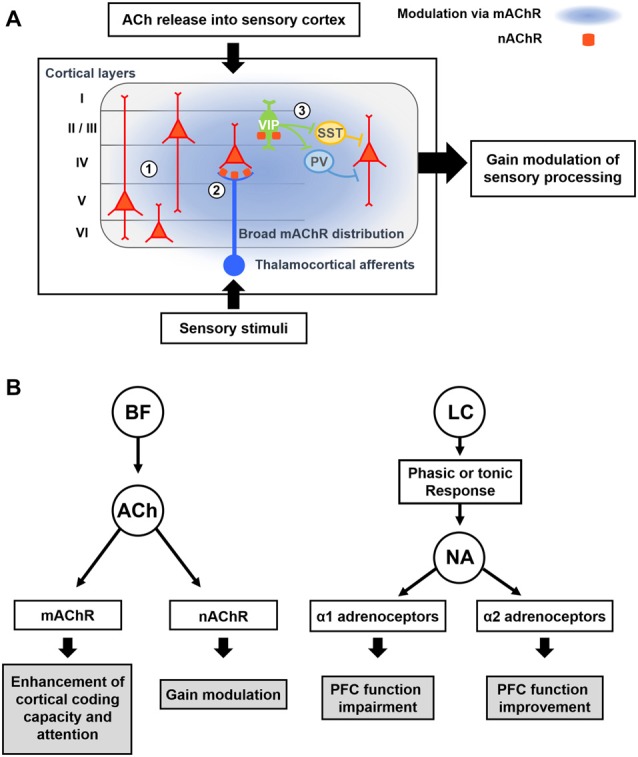
Modulatory effects of acetylcholine (ACh) and Noradrenaline (NA) on cortical processing through different types of receptors.** (A)** Schematic description of cholinergic modulation in the primary sensory cortex. ① Broad distribution of metabotropic ACh receptor (mAChR) mediates modulation of both excitatory and inhibitory neurons across the layers (Alitto and Dan, 2013). ② nicotinic AChRs (nAChRs) are expressed in the thalamocortical axon terminals, and cholinergic activation of them causes increase in sensory responses of neurons in the input layer (Lavine et al., 1997; Metherate, 2004; Disney et al., 2007). ③ nAChRs are expressed in the vasoactive intestinal peptide-positive (VIP+) neurons, which elicit disinhibition of pyramidal neurons by inhibiting SST+ or PV+ inhibitory neurons (Harris and Mrsic-Flogel, 2013; Lee et al., 2013; Pi et al., 2013). Cholinergic activation of VIP+ neurons can increase the sensory gain via this disinhibitory circuit (Porter et al., 1999; Fu et al., 2014). **(B)** Modulatory effects of ACh and NA. (*Left*) ACh released from the BF enhances cortical processing via both mAChRs and nAChRs. Activation of mAChR enhances cortical coding capacity of sensory stimulus (Goard and Dan, 2009), while activation of nAChRs increases the sensory gain in the visual cortex (Metherate, 2004; Disney et al., 2007). (*Right*) Two distinct modes of the noradrenergic modulation. LC neurons show either phasic or tonic activity patterns depending on the states. Sensory stimuli evoke phasic responses of NA neurons whereas stressful stimuli evoke both phasic and tonic responses (Aston-Jones et al., 1999). When the animal shows focused attention or engages in the task, the NA neurons show phasic activity. Conversely, NA neurons show tonic responses when the animal is distracted or shows flexible behaviors. The amount of NA released from noradrenergic neurons determines the activation of different types of adrenoceptors, which modulate the PFC function in an opposite manner (Ramos and Arnsten, 2007). A moderate amount of the NA preferentially activates the α2 adrenoceptor, which has a higher binding affinity for the NA, and improves the PFC function such as working memory and focused attention (Li and Mei, 1994; Li et al., 1999). In contrast, when a higher concentration of NA is released, the α1 adrenoceptors are activated as well, which can lead to the impairment of PFC function (Arnsten et al., 1999; Mao et al., 1999; Ramos and Arnsten, 2007).

Transmission selectivity in the cortical space is also tightly related to the distribution of ACh receptors (AChRs; Figure 3A). The metabotropic AChR (mAChR) has five subtypes, m1–5. The m1, m3 and m5 subtypes are coupled with Gq proteins that trigger the inositol phosphate pathway. The m2 and m4 subtypes are coupled with Gi proteins, which suppress adenylyl cyclase activities (Felder, 1995). The m1 and m2 subtypes are found at the cholinergic synapses as well as the non-cholinergic synapses (Mrzljak et al., 1993). Moreover, the m1 AChR subtype is found over the somatodendritic membrane (Yamasaki et al., 2010). These expression patterns suggest that volume transmission of ACh might occur through the m1 and m2 receptors. In addition, the m2 and m4 receptors are found in presynaptic terminals and work as autoreceptors. These autoreceptors can regulate the release of ACh from presynaptic terminals (Zhang et al., 2002).

The nicotinic AChRs (nAChRs) are ionotropic receptors that can generate fast excitatory postsynaptic potentials. In the macaque primary visual cortex (V1), nAChRs are found in thalamic axons of excitatory neurons in layer 4c as well as in inhibitory interneurons (Disney et al., 2007). Treatment of nicotine into V1 can suppress visual responses of neurons other than the layer 4c neurons receiving thalamic inputs, and this effect can enhance visual gain and reduce the detection threshold of layer 4c neurons. Similarly, in the rat cortex, nAChRs have been found in axon terminals of thalamic afferents (Lavine et al., 1997; Metherate, 2004) and a subset of GABAergic neurons including the vasoactive intestinal peptide-positive (VIP+) GABAergic neurons (Porter et al., 1999). Electrical stimulation of the BF can facilitate thalamocortical transmission (Metherate and Ashe, 1993) and activate VIP+ neurons in the cortex (Alitto and Dan, 2013). VIP+ GABAergic neurons mainly inhibit other types of GABAergic neurons in the cortex (Lee et al., 2013; Pi et al., 2013). Therefore, cholinergic activation of VIP+ neurons can induce disinhibition on pyramidal neurons, which can mediate the locomotion-induced enhancement in visual responses and orientation selectivity in V1 neurons (Fu et al., 2014). Moreover, activation of BF cholinergic neurons causes disinhibition in the auditory and somatosensory cortices as well (Froemke et al., 2007; Kruglikov and Rudy, 2008; Letzkus et al., 2011), suggesting disinhibition is a general feature of cholinergic modulation in the cortex. In the auditory cortex, however, GABAergic neurons than VIP+ interneurons are also found to receive mono-synaptic inputs from the BF cholinergic neurons (Letzkus et al., 2011; Nelson and Mooney, 2016). Furthermore, parallel modulation of all types of GABAergic neurons by cholinergic inputs can be critical for the context-dependent cortical processing (Kuchibhotla et al., 2017). Future studies are required to fully understand function of cell-type-specific cholinergic modulation in other cortical areas.

## Noradrenaline (Norepinephrine)

### Anatomical Organization of the Noradrenergic System

Noradrenaline (NA) regulates a number of brain functions, such as sleep/wakefulness and attention, and it has the potential of modulating wide brain regions including the hippocampus, the amygdala, the thalamus, and the cerebral cortex (Foote et al., 1983; Sara and Bouret, 2012). The locus coeruleus (LC), which is located in the brainstem, is the exclusive source of NA in the central nervous system (CNS; Dahlstroem and Fuxe, 1964; Swanson, 1976). The LC is composed predominantly by a population (90%) of noradrenergic neurons with a small proportion of non-noradrenergic cells such as serotonergic and GABAergic neurons (Iijima, 1989, 1993). Several studies have reported that noradrenergic neurons co-express neuropeptides such as galanin and neuropeptides Y (NPY; Olpe and Steinmann, 1991; Schwarz and Luo, 2015). Despite the small numbers of noradrenergic neurons (~1500 and ~5000 per each hemisphere in mouse and monkey, respectively) and tiny size of the LC, most of the cortical regions are known to receive extensive noradrenergic innervations from the LC (Sturrock and Rao, 1985; Sara, 2009). Thus, the noradrenergic neurons in the LC naturally have a higher potential of divergence in their projection.

Early anatomical studies identified the axonal projections of the LC neurons by injecting radioisotopes or anterograde tracers into the LC and via immunostaining of the noradrenergic fibers against the dopamine-beta-hydroxylase (DBH; Morrison et al., 1978, 1982; Verney et al., 1984; Audet et al., 1988; Doucet et al., 1988). Axon terminals of the LC neurons are observed ubiquitously across the cerebral cortex (Jones and Moore, 1977; Jones et al., 1977; Jones and Yang, 1985; Loughlin et al., 1986; Samuels and Szabadi, 2008). Interestingly, there is regional variation of noradrenergic fiber densities among the different cortical areas. The fiber density of noradrenergic neurons is higher in the frontal cortex than in the motor and the sensory cortex (Agster et al., 2013). In addition, within the PFC sub-regions, the fiber density is not homogeneous (Lewis and Morrison, 1989). Thus, despite widespread cortical distribution of noradrenergic axon terminals, some cortical regions might receive denser noradrenergic inputs and show stronger modulation by NA.

Does a single LC noradrenergic neuron project to multiple cortical areas? Retrograde tracing studies have shown divergence of noradrenergic efferent fibers and proved that substantial amounts of LC noradrenergic axons bifurcate to different cortical regions (Figure 2; Swanson and Hartman, 1975; Porrino and Goldman-Rakic, 1982; Kim et al., 2016). Recent viral tracing results also confirmed diverging projections of the noradrenergic LC neurons (Schwarz et al., 2015). In contrast, Waterhouse et al. (1990) proposed the possibility of selective projections of LC neurons. By injecting retrograde tracers into different sub-regions of the PFC, they found only 4% of the LC neurons send diverging projections into the PFC sub-regions (Chandler and Waterhouse, 2012; Chandler et al., 2013). Taken together, the presence of widespread and divergent axonal projections suggest LC noradrenergic neurons can play an important role in the global regulation of cortical activities, such as sleep, wakefulness, and arousal. Conversely, LC projection to the confined cortical area proposes a potential role in selective attention. In summary, the anatomy of LC noradrenergic neurons shows a heterogeneous nature including both divergent and selective projections (Kebschull et al., 2016), which implies a functional diversity and complexity.

### Noradrenergic Transmission in the Cerebral Cortex

Efferent noradrenergic axon terminals arising from the LC release NA, which binds to adrenergic receptors composed of the α1, α2 and β receptor families (Molinoff, 1984; Ramos and Arnsten, 2007). These receptors co-exist across the cortical areas, showing overlapping expression patterns. These receptor families, which are all classified as G-protein coupled receptors (O’Donnell et al., 2012), have several subtypes showing distinct expression patterns in the CNS (Ramos and Arnsten, 2007). First, the α1 family of receptors show an intermediate binding affinity to NA and are coupled to Gq proteins (Hieble et al., 1995; Sirviö and MacDonald, 1999). There are three subtypes of α1 in the cortex: α1A, B, D receptors. Among them, the α1D receptors show the highest cortical expression with laminar preference of the superficial layer (Goldman-Rakic et al., 1990; Pieribone et al., 1994). Second, the α2 receptors have the highest binding affinity to NA and are coupled to the Gi proteins (Ramos and Arnsten, 2007). Among the α2 receptor subtypes, including α2A~C receptors, the α2A receptor is the most abundant in the cerebral cortex (Scheinin et al., 1994). This receptor is also expressed more in the superficial layer (Goldman-Rakic et al., 1990). Finally, β adrenergic receptors are coupled to Gs proteins and comprise the β1~3 subtypes. They have the lowest binding affinity to NA (Minneman et al., 1981). The β1 and β2 subtypes show the most prevalent expression in the cortex, mostly in layer 4. Taken together, different adrenergic receptors recruit different intracellular signaling pathways, which can result in distinct modulation effects on the cortical neurons.

In addition to the classical synaptic transmission, NA is also released non-synaptically and diffuses across the broad extracellular space (Agnati et al., 1995, 2010). This volume transmission of NA can modulate target neurons in the broader area for a longer time (Sara, 2009; O’Donnell et al., 2012). The α2A and β receptors are found in both the dendritic spines and non-synaptic areas including the axons and the dendritic shafts, supporting the non-synaptic volume transmission of NA (Herkenham, 1987; Nicholas et al., 1993; Aoki et al., 1998). The axonal expression of α2A and β receptors suggests their function as an autoreceptor or a heteroreceptor that regulates the release of neurotransmitters including NA itself (Starke, 2001). Adrenergic receptors expressed in the dendritic shafts often do not overlap with the noradrenergic axonal fibers (Seguela et al., 1990). These receptors might be activated by the diffused NA from the releasing terminals (Vizi et al., 2004). Future studies are required to understand the function of these non-synaptic NA receptors within the complicated cortical circuits *in vivo*.

Since α1 and α2 adrenergic receptors have different levels of affinity to NA, the local concentration of NA released from noradrenergic neurons can activate these receptors differentially (Figure 3B). More α1 receptors are activated at higher concentration of NA, while mild concentrations of NA preferentially activates α2 receptors (Ramos and Arnsten, 2007). As α1 adrenoceptors are excitatory, whereas α2 adrenoceptors are inhibitory and suppress the synaptic release (Szabadi, 2013), activation of different NA receptors in the cortex can induce quite opposite modulatory effects. Accordingly, when the NA neurons show high levels of tonic and phasic firing activity, such as when the animal is under strong stressors, high levels of NA can be released in the cortex. This can activate α1 adrenergic receptors, which can lead to the impairment of cortical function (Arnsten et al., 1999). Conversely, when NA neurons show moderate activity in normal conditions, α2 receptors are preferentially activated and cortical function can be improved (Figure 3B; Arnsten and Li, 2005; Arnsten, 2009).

## Beyond the Selective Projections: Complete Understanding of the Input-Output Circuits

Although it has been known that BF cholinergic cells and LC noradrenergic cells receive inputs from diverse regions and show differential projection patterns, the exact input-output relation of each system has been ambiguous. As we discussed above, BF cholinergic neurons show selective projections to the cortex. If these selective projections indeed modulate the cortical sub-regions independently in the intact and naturally functioning brain, the inputs to BF cholinergic neurons must be segregated and activated in an output-specific manner. Supporting this idea, cortical inputs to the BF are segregated, as BF neurons show selective responses to electrical stimulation of the PFC (Golmayo et al., 2003). In this study, only 42% of recorded BF cells responded to electrical stimulation of the cingulate cortex and only 33% of them responded to that of the secondary motor cortex, whereas the rest of them responded to stimulation of both. Although it has been reported that most of the BF neurons that receive the PFC inputs are GABAergic (Zaborszky et al., 1997), the local inhibition might control the cholinergic output selectively (Xu et al., 2015). Other important inputs to the BF are the neuromodulatory neurons. The dopaminergic neurons from the VTA (Zaborszky et al., 1997) and serotonergic neurons in the dorsal raphe nucleus have been found to project to the BF (Jones and Cuello, 1989). LC noradrenergic neurons also show strong projection to the BF (Espana and Berridge, 2006). Neurons in the striatum and the amygdala project to the BF as well (Hu et al., 2016; Gielow and Zaborszky, 2017). Future studies are required to determine whether these neuromodulatory projections are selective into the BF.

**Figure 4:**
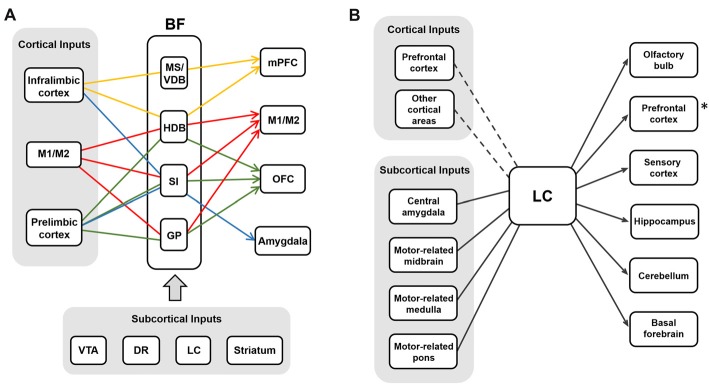
The input-output circuits of the BF-ACh neurons and the LC-NA neurons.** (A)** BF cholinergic neurons project selectively to different brain regions based on their input regions (Gielow and Zaborszky, 2017). Each color represents the selective input-output relationship of the BF ACh neurons. Cortical and subcortical inputs are shown in the gray boxes. **(B)** LC noradrenergic neurons receive converging inputs and show diverging projections to various brain areas (Schwarz et al., 2015). The PFC is one of the strong cortical inputs, although the cortical afferent to the LC is relatively weaker than the subcortical afferent. The asterisk (*) refers the Figure 2B where the noradrenergic projection to the PFC is selective rather than diverging in rats (Chandler et al., 2013).

Similar to BF neurons, LC noradrenergic neurons receive converging inputs from various brain areas including the cortex, the amygdala, the hypothalamus, the thalamus, the pons, the medulla and the cerebellum (Aston-Jones and Cohen, 2005a; Szabadi, 2013; Schwarz et al., 2015). Recent studies using cell-type specific and monosynaptic retrograde tracing with pseudo-typed rabies virus investigated the input-output relations of cholinergic neurons in the BF (Gielow and Zaborszky, 2017) and noradrenergic neurons in the LC (Schwarz et al., 2015). Interestingly, these studies have shown that BF cholinergic cells receive selective inputs depending on their projection regions, whereas LC noradrenergic cells receive converging inputs that are not segregated to the neurons projecting to different areas (Figure 4). This implies BF cholinergic neurons may be able to work as separate streams depending on the input conditions and the demands of selective cholinergic modulation. The LC noradrenergic neurons receive converging inputs and send diverging projections to the cortex, and this might be able to mediate the holistic modulation of the brain during arousal and the switch from sleep to wakefulness.

One interesting characteristic of the BF and the LC circuits is the unidirectional projection of LC noradrenergic neurons to the BF. In the BF, cholinergic neurons express both α1 and β1 adrenoceptors whereas GABAergic neurons express α2 adrenoceptors (Manns et al., 2003; Szabadi, 2013; Schwarz and Luo, 2015). The adrenergic activation of α1 receptors activates the neurons expressing the receptors, while the activation of α2 adrenoceptors suppresses the neurons. Thus, the net modulation effect by noradrenergic inputs to the BF is the enhancement of ACh release in the cortex (Schwarz and Luo, 2015). As the LC noradrenergic neurons play crucial roles in changing the global brain states, the BF cholinergic neurons receiving these noradrenergic inputs might contribute to the changes in global brain states. Supporting this, both the LC noradrenergic neurons and the BF cholinergic neurons are most active during wakefulness and play critical roles in controlling sleep (Carter et al., 2010; Xu et al., 2015). Further studies are required to understand how selective the LC projections are into the BF and how these two distinct neuromodulators work together throughout the cortex during the sleep-wake cycle.

## Functional Comparison of the Cholinergic and the Noradrenergic Projections

### Attention

Visual attention is an important brain function that requires modulation of the sensory cortex. The ACh is proposed as one of the key modulators for modulation of the cortex during attention. Indeed, lesion on cholinergic neurons in the BF caused impairments of selective attention in animals performing tasks (Voytko et al., 1994; McGaughy et al., 2002). Treatments with cholinergic agonists or antagonists enhance or suppress visual attention in humans (Furey et al., 2008). In macaque monkeys, spatial attention induces ACh release in the V1, and this leads to activation of mAChRs that is critical for visual attention (Herrero et al., 2008; Thienel et al., 2009). Electrical stimulation of the BF can enhance information processing of V1 neurons via activation of mAChRs (Goard and Dan, 2009). Furthermore, optogenetic activation of either cholinergic neurons or cholinergic fibers in the V1 improves the discrimination of low-contrast visual stimuli in mice (Pinto et al., 2013). Thus, cholinergic modulation of the visual cortex is critical for the animal to increase spatial attention to the important visual stimuli in the environment.

In addition to the sensory cortices, the PFC is also known to be modulated by ACh during attention. In trials of a cued-appetitive response task with the reward delivered randomly into one of two reward ports, the ACh concentration is increased in the mPFC when an animals shows sustained attention (Parikh et al., 2007). Thus, unlike visual attention that modulates the visual cortex, sustained attention requires cholinergic modulation of the PFC. It is still unknown whether these two types of attentional modulation are mediated by the segregated BF cholinergic neurons. Similar to cholinergic modulation, several studies have shown that noradrenergic modulation is also important for attention (Smith and Nutt, 1996; Aston-Jones et al., 1999; Aston-Jones and Cohen, 2005b). Inflicting a lesion on the dorsal noradrenergic bundle that induces NA depletion in the neocortex and the hippocampus causes clear behavioral deficits in rats performing 5-choice serial reaction tasks, which are known to require attention in rats (Carli et al., 1983). Rats with the lesion show a decrease in the choice accuracy and an increase in trial omissions. When the firing activity of LC neurons was measured in animals performing the attentional tasks, the LC neurons show higher responses to the task-relevant cues, while weakly or not responding to the distractors (Usher et al., 1999). Thus, both BF cholinergic neurons and LC noradrenergic neurons are active and important for the attentional modulation of the cortex.

Interestingly, the LC neurons exhibit phasic firing activity in most of the correct trials, whereas they show tonic discharges during the incorrect trials when the rat performs the attention tasks (Usher et al., 1999). Based on these results, the “inverted U-shape” response pattern of LC neurons has been proposed on the relationship between LC neuron activity and the level of attention: when the animal is more attentive, LC neurons show phasic activity, and when they are less attentive and possibly aroused, LC neurons maintain tonic firing activity (Aston-Jones et al., 1994, 1997; Rajkowski et al., 1994). Future studies are required to fully understand how the firing pattern of LC noradrenergic neurons determines the mode of cortical modulation by recruiting different adrenoceptors in the cortex (Carter et al., 2010).

### Reinforcement

Recent studies have proposed that BF cholinergic neurons might be more active during reinforcement rather than during attention (Hangya et al., 2015). The BF neurons that show correlated firing activity with sustained attention in a trial-to-trial manner are mainly identified as non-cholinergic neurons (Nguyen and Lin, 2014; Hangya et al., 2015). Furthermore, the optogenetically identified cholinergic neurons show strong responses to the reinforcement (either the reward or the punishment) in the same animal performing attentional tasks (Hangya et al., 2015). In this study, both the HDB and NB cholinergic neurons show stronger responses to the negative reinforcements than to the positive ones. In another study, it has also been shown that BF cholinergic projections to the V1 is necessary for the acquisition of reward timing in behaving rats, supporting the idea that BF cholinergic neurons are strongly involved in delivering the reinforcement signal to the cortex (Chubykin et al., 2013). Interestingly, the LC neurons also show strong responses to the reinforcement (Bouret and Sara, 2004). Thus, responses of cholinergic neurons to reinforcements can be originated from the LC noradrenergic neurons that project to the BF cholinergic neurons (Espana and Berridge, 2006). Otherwise, a common input such as dopaminergic projections to both BF and LC might activate them together when reinforcements are presented (Ornstein et al., 1987; Jones and Cuello, 1989; Woolf, 1991; Sara, 2009). Many behavioral experiments, however, use rewards or punishments to train animals, and these reinforcements naturally make animals pay more attention to the relevant sensory stimuli and facilitate their learning. Thus, it is difficult to dissociate the reinforcement-related activity from the attention-related activity in many brain areas (Maunsell, 2004), and this can be true in neuromodulatory systems. It is also possible that a subset of cholinergic or noradrenergic neurons are more activated by external stimuli such as rewards or punishments rather than by changes in internal states such as attention. This needs to be clearly understood in future studies.

### Learning and Memory

A large body of literature has shown that there is a significant correlation between Alzheimer’s disease and degeneration of cholinergic fibers in the forebrain (Whitehouse et al., 1981; Coyle et al., 1983; Terry and Buccafusco, 2003). Indeed, cholinergic modulation of cortex and hippocampus is well-known to be critical for learning and memory in mammals (Power et al., 2003). Early studies have shown that electrical stimulation of the NB paired with tone stimuli changes the cortical map and reorganizes the receptive field structures in the auditory cortex (Bakin and Weinberger, 1996; Kilgard and Merzenich, 1998). In more recent studies, Froemke et al. (2007, 2013) have further shown that this network-level plasticity is clearly linked to synaptic level plasticity in the auditory cortex as well as perceptual improvements with learning. Like other neuromodulators, cholinergic modulation induces synaptic plasticity via activating secondary messenger systems (Seol et al., 2007) and ACh and NA are key neuromodulators that induce long-term synaptic modification in the visual cortex during ocular dominance plasticity (Bear and Singer, 1986). Thus, synaptic plasticity induced by neuromodulatory inputs to the cortex might be a common underlying mechanism for different forms of perceptual learning.

In addition to the sensory cortex, cholinergic modulation of the PFC is important for working memory. Injection of mAChR antagonist scopolamine into the ACC and the prelimbic cortex (PL) of rats induces impairment of the working memory even though the rats detected the visual signal correctly (Chudasama et al., 2004). Cholinergic projection from the medial septum to the hippocampus releases ACh in the hippocampus and modulates the network to a state of memory consolidation (Hasselmo, 1999). The m2 and m4 AChR knock-out mice show dysregulation of the ACh release in the hippocampus and impairments in the cognitive behavior (Tzavara et al., 2003). The hippocampus receives cholinergic inputs mainly from the VDB and MS of the BF (Nyakas et al., 1987), and the lesion of the MS cholinergic neurons induces memory deficits in rats performing the radial-arm maze task with random delays. It has also been shown that the theta oscillation in the hippocampus is important for learning and memory, and this theta oscillation is mainly induced by the cholinergic efferent to the hippocampus (Buzsáki, 2002).

Sara and colleagues have shown the role of noradrenergic modulation of the cortex during learning and memory (Sara, 2009; Sara and Bouret, 2012). They found that local inactivation of the β adrenergic receptors in the PL of the rats after the operant learning induces memory deficits, suggesting that the noradrenergic modulation of the PL is necessary for memory consolidation (Sara et al., 1999; Tronel et al., 2004). Supporting this idea, they measured the extracellular NA level in the PL and found that it is increased in the learned animal (Tronel et al., 2004). Other studies have reported that working memory can be modulated by the NA in the PFC. Local infusion of the α2 agonist into the PFC of the rat enhances its performance in the working-memory task (Tanila et al., 1996), whereas microinjection of the α2 antagonist into the dorsolateral PFC disrupts spatial working memory of the monkey (Li and Mei, 1994). Conversely, the local infusion of the α1 adrenoceptor agonist into the PFC impairs spatial working memory in both monkeys and rats (Arnsten et al., 1999; Mao et al., 1999). Under the same condition, pretreatment of the α1 receptor antagonist rescues the impairment, indicating the specific role of the α1 receptor (Mao et al., 1999). Collectively, the noradrenergic system plays a crucial role in learning and memory, and different types of adrenergic receptors show opposite functions in it. In particular, the α1 receptor impairs working memory, whereas the α2 receptor enhances it (Arnsten et al., 1998). Interestingly, unlike the working memory task, activation of the α1 receptor is required for the attentional set shifting task (Lapiz and Morilak, 2006). Working memory requires the animal to retain the information just acquired, whereas attentional set shifting requires the animal to abandon the current information and move on to the novel sensory information (Lapiz and Morilak, 2006). Thus, the activation of the α1 receptors by the high level of the NA might not be always negative and necessary for the better performance depending on the cognitive demand of the tasks. It will be interesting to study whether the selective projection of the LC noradrenergic neurons to the PFC plays any role in these functions (Chandler and Waterhouse, 2012).

### Sleep and Wakefulness (Global Brain States)

Although the cholinergic neurons show selective innervation to the cortex, it has also been known that the cholinergic neurons can be involved in modulation of the global brain states during sleep. The BF cholinergic neurons are highly active during wakefulness and paradoxical sleep but show low activity during slow-wave sleep (Lee et al., 2005). Burst firing activity of cholinergic neurons induces broad theta oscillations in the hippocampus and the cortex (Lee et al., 2005). A recent study showed that the cholinergic neurons are active during wakefulness and rapid-eye-movement sleep in mice, and showed that artificial activation of cholinergic neurons in the BF induces the transition from sleep to wakefulness (Xu et al., 2015). However, it is still unclear whether any specific population of corticopetal BF cholinergic neurons is responsible for this induction of wakefulness. Furthermore, as shown in human studies, it might be critical to maintain the reduced level of ACh during slow-wave sleep for the consolidation of the declarative memory in rodents (Gais and Born, 2004). It will be interesting to examine whether activity of the BF cholinergic neurons during sleep is important for memory consolidation (Power et al., 2003).

The LC noradrenergic system is also known to be involved in controlling sleep (Aston-Jones and Bloom, 1981; Berridge and Waterhouse, 2003; Atzori et al., 2016). The LC neurons show less firing activity during non-rapid eye movement (NREM) sleep and become almost silent during rapid eye movement (REM) sleep. During wakefulness, the LC neurons show either tonic firing activity at 1–3 Hz in quiet wakefulness or phasic firing activity at 8–10 Hz bursts when the animal receives salient stimuli (Hobson et al., 1975; Foote et al., 1980; Aston-Jones and Bloom, 1981; Rasmussen et al., 1986; Eschenko et al., 2012). The transition of the LC firing activity precedes the switch in the behavioral states, and the pharmacological administration of α1 and β receptor antagonists elicit an increase in slow-wave activity and a reduction in behavioral activities (Schmeichel and Berridge, 2013). A recent study showed that optogenetic activation of LC noradrenergic neurons at phasic (10 Hz) and at tonic (3 Hz) activity induces immediate sleep-to-wakefulness transitions, whereas inactivation of these neurons cause the reduction of wakefulness (Carter et al., 2010). Therefore, the activity of the LC noradrenergic neurons is critical for the induction and maintenance of wakefulness. Furthermore, many studies have shown a strong correlation between the LC activity and pupil size, which represents the level of arousal in an awake animal (Aston-Jones and Cohen, 2005b; Murphy et al., 2014; Joshi et al., 2016). Interestingly, the rapid pupil dilation is caused by phasic activity of LC noradrenergic neurons, and long-lasting dilation of the pupil during locomotion is more correlated with sustained activity of cholinergic neurons (Reimer et al., 2016). These results indicate that elevated activity in the LC noradrenergic neurons can mediate global brain state transitions to wakefulness and rapid arousal. As discussed earlier, the diverging and extensive innervation of the LC noradrenergic neurons to the cortex may support this function.

## Conclusion

The BF cholinergic and LC noradrenergic systems share common features: broad cortical innervations and regulation of cognitive functions such as arousal, attention, learning, and sleep. However, they clearly show distinct anatomical and physiological characteristics. First, the BF is constructed with multiple sub-nuclei, which project to distinct regions in the brain (Figure 1A). The LC, however, is a small nucleus with noradrenergic neurons that project to wider brain areas (Figure 1B). Thus, the level of divergence of these projections must be different between the systems. Second, the topographic distribution of the axonal projections in the cortex is different between these two systems. Although both neuromodulatory systems modulate the sensory cortices (Figure 1; Waterhouse et al., 1990; McLean and Waterhouse, 1994; Manunta and Edeline, 1997; Disney et al., 2007; Goard and Dan, 2009; Pinto et al., 2013; Fu et al., 2014), the cholinergic neurons show selective projections, whereas the noradrenergic neurons show diverging projections to the sensory cortex (Figure 2A; Chaves-Coira et al., 2016; Kim et al., 2016). Their projection to the PFC shows opposite patterns (Figure 2B; Chandler and Waterhouse, 2012; Chandler et al., 2013). Third, the cell types and receptor types that receive the modulation is distinct between the systems. Downstream signaling pathways can be either excitatory or inhibitory depending on the receptor types. The activity pattern of the cholinergic and the noradrenergic neurons must be considered to fully understand the level of modulation in the cortex (Figure 3). Finally, the inputs to the BF and the LC can show different levels of selectivity (Figure 4). Recent studies have begun to map the whole-brain inputs to the neuromodulatory systems (Schwarz et al., 2015; Gielow and Zaborszky, 2017). To fully understand the function of these neuromodulatory projections in the cortex, it is necessary to examine how the selective inputs and their outputs are linked together to induce a particular activity pattern in the population of the cholinergic and noradrenergic neurons and how they exert specific brain functions that require their neuromodulation: attention, arousal, learning and transition in the global brain states.

## Author Contributions

H-JR designed the figures and wrote the manuscript. J-HK wrote the manuscript. S-HL conceived the contents and wrote the manuscript. All authors discussed the contents and revised the manuscript.

## Conflict of Interest Statement

The authors declare that the research was conducted in the absence of any commercial or financial relationships that could be construed as a potential conflict of interest.
